# Elucidation of the heme active site electronic structure affecting the unprecedented nitrite dismutase activity of the ferriheme *b* proteins, the nitrophorins†
†Electronic supplementary information (ESI) available: Figures and tables. See DOI: 10.1039/c6sc01019a


**DOI:** 10.1039/c6sc01019a

**Published:** 2016-04-25

**Authors:** Chunmao He, Hideaki Ogata, Wolfgang Lubitz

**Affiliations:** a Max Planck Institute for Chemical Energy Conversion , Stiftstrasse 34-36 , D-45470 , Mülheim an der Ruhr , Germany . Email: chunmao82@gmail.com ; Email: wolfgang.lubitz@cec.mpg.de

## Abstract

Nitrophorins (NPs) catalyze the nitrite dismutation reaction that is unprecedented in ferriheme proteins. Despite progress in studying the reaction mechanism, fundamental issues regarding the correlation of the structural features with the nitrite dismutase activity of NPs remain elusive. On the other hand, it has been shown that the nitrite complexes of NPs are unique among those of the ferriheme proteins since some of their electron paramagnetic resonance (EPR) spectra show significant highly anisotropic low spin (HALS) signals with large *g*_max_ values over 3.2. The origin of HALS signals in ferriheme proteins or models is not well understood, especially in cases where axial ligands other than histidine are present. In this study several mutations were introduced in NP4. The related nitrite coordination and dismutation reaction were investigated. As a result, the EPR spectra of the NP–nitrite complexes were found to be tightly correlated with the extent of heme ruffling and protonation state of the proximal His ligand—dictated by an extended H-bonding network at the heme active site. Furthermore, it is established that the two factors are essential in determining the nitrite dismutase activity of NPs. These results may provide a valuable guide for identifying or designing novel heme proteins with similar activity.

## Abstract 2

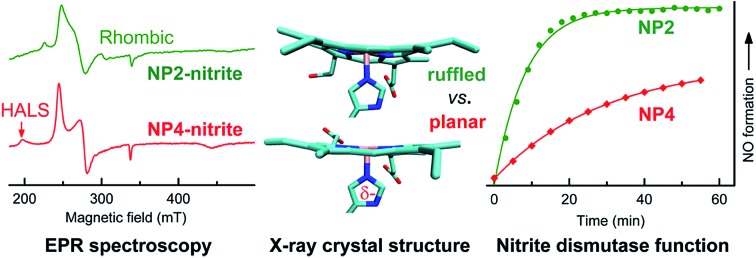
A highly ruffled heme conformation and an extended H-bonding network at the heme active site are the key factors facilitating the unprecedented nitrite dismutase activity of the nitrophorins.

## Introduction

Nitrite is a major player in the nitrogen biogeochemical cycle, the biological fate of which is largely determined by a number of metalloproteins.^1^ Heme proteins are among the most important regulators of nitrite, catalyzing a variety of reactions—dictated by the heme core,^1^,^2^ *e.g.* the cytochrome *c* nitrite reductase (*cc*NiR),^3^ the assimilatory NiR (aNiR),^4^ and the two-domain protein cytochrome *cd*_1_NiR.^5^ Recently, nitrite has been suggested as a viable source of nitric oxide (NO) in humans, thereby, it is involved in the signaling pathways.^6^ The reduction of nitrite to NO is facilitated by a number of human heme *b* proteins, including hemoglobin (Hb) and myoglobin (Mb).^7^,^8^ It is worth noting that, although the detailed reaction mechanism remains elusive,^7^,^9^,^10^ all the functional heme proteins react at a ferrous oxidation state in the reduction of nitrite to NO.

Another heme *b* containing protein class with a novel type of nitrite reaction is the nitrophorins (NPs) that originate from the saliva of the blood feeding insect *Rhodnius prolixus*.^11^,^12^ At least five isoforms appear concurrently in the insect saliva, termed NP1–4 and NP7.^13^,^14^ The biological function of the ferriheme *b* NPs has been established as an NO transporter. Interestingly, NPs were found to be able to convert nitrite into NO *via* a ‘nitrite-only’ mechanism with the stoichiometry of the nitrite disproportionation reaction.^15^

1 3NO_2_^–^ + 2H^+^ → 2NO + NO_3_^–^ + H_2_O

The ability of NPs to catalyze this reaction at neutral pH—that leads to the electron paramagnetic resonance (EPR)-silent {FeNO}^6^ complex according to the notation of Enemark and Feltham^16^—is unprecedented among any other hemes. The NPs are therefore classified as nitrite dismutases (EC 1.7.6.1).^17^

**Scheme 1:**
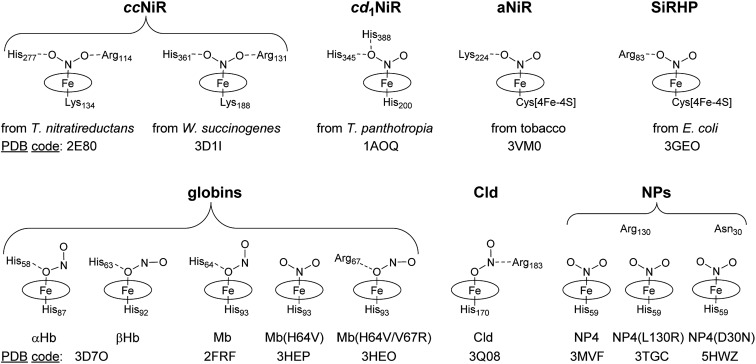
Comparison of the nitrite binding modes in selected ferriheme protein complexes. (SiRHP, sulfite reductase hemoprotein).

The coordination chemistry of heme iron with nitrite is important for understanding the catalytic mechanism of the enabling heme proteins. Several crystal structures of ferriheme proteins in complex with NO_2_^–^ were solved and the related heme cores are summarized in Scheme 1. Similar to the model hemes,^18^,^19^ all structures of the NiR complexes show η^1^-N (nitro) coordination.^4^,^5^,^20^–^22^ In all the cases of NiR, the ligand is further bound through H-bonding to Arg, His, or Lys side-chains. With the advent of the metHb[NO_2_^–^]^23^ and metMb[NO_2_^–^] structures,^24^ the first examples of stable η^1^-O coordinate heme nitrito complexes were reported (Scheme 1). Similarly, the nitrite was held by H-bonding of Fe–O_nitrite_ with the distal His and it was found that the distal H-bonding residues determine the NO_2_^–^ coordination mode.^25^ Like the globins, the heme *b* protein chlorite dismutase (Cld)–NO_2_^–^ has an η^1^-O nitrite ligand that is further bound to Arg183.^26^ It is generally believed that the bound NO_2_^–^ is activated through H-bonding to the charged residues at the active site during turnover.^1^

In contrast to all reported crystal structures, the NO_2_^–^ ligand in NP4[NO_2_^–^] is not H-bonded to any residue side-chain while the protein forms a classical N-bound nitro complex (Scheme 1).^27^ In probing the determining factors of the NO_2_^–^ binding mode in NP4, the heme distal pocket variant NP4(L130R) was produced, the structure of which is almost the same as that of the wild-type (wt).^28^ Interestingly, NO_2_^–^ remains N-bound in this variant, demonstrating that, unlike the metMb[NO_2_^–^],^25^ the presence of an H-bond donating residue is not sufficient to change the mode of NO_2_^–^ coordination in NP4.^29^ Thus, the question remains—how are the nitrite ligand orientation and the nitrite reductase activity determined in NPs?

**Fig. 1:**
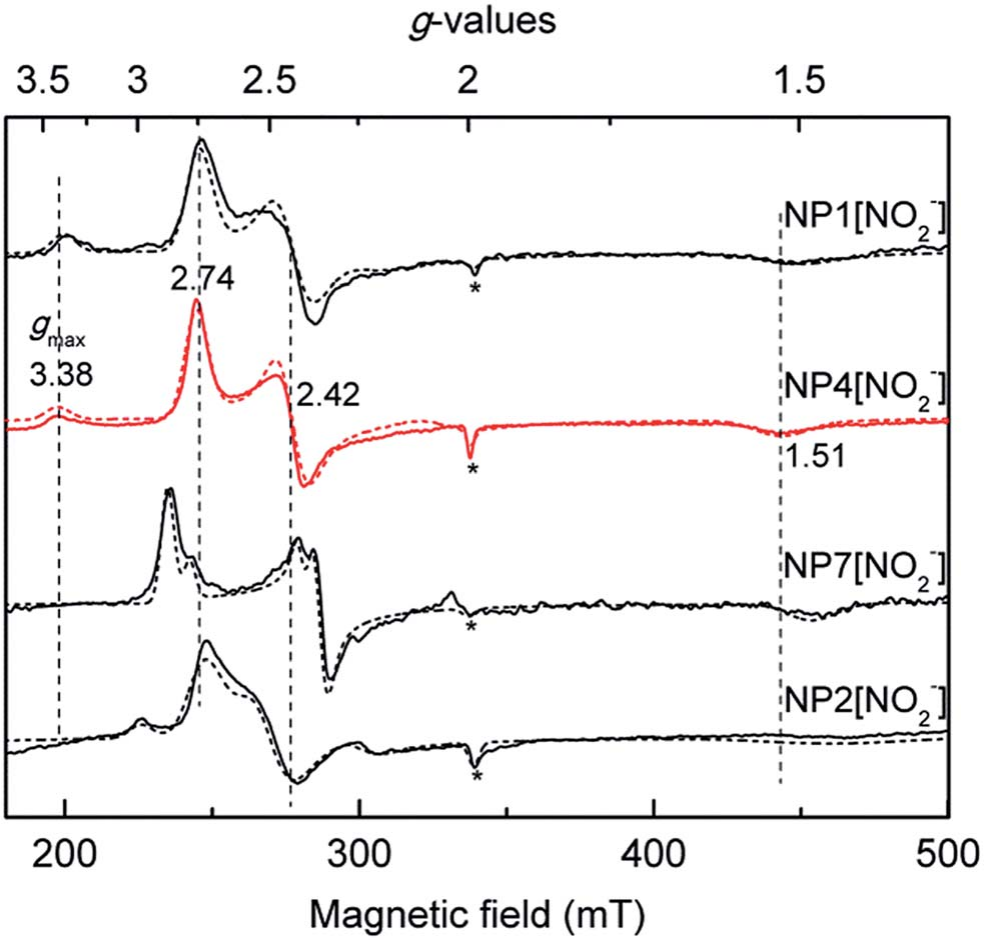
Comparison of the X-band EPR spectra of the nitrite complexes of different NPs recorded at 10 K, with the simulated spectra shown in dashed lines (see Fig. S2† for *g*-values used for the simulations). For comparison, the positions of the *g*-values of NP4 are indicated with dashed vertical lines. Note that in all the cases a small fraction of the high-spin signal was observed (*), possibly due to a water-bound ferriheme complex.

EPR spectroscopy has been widely applied in probing the electronic configuration of the ferriheme–NO_2_^–^ complexes.^30^ In most of these complexes, two sets of *g*-values were obtained in X-band EPR spectra that reflect low-spin (LS) electron configurations (*S* = 1/2).^27^ In the cases of metMb[NO_2_^–^] and metHb[NO_2_^–^], several suggestions were made related to the origin of the two species, for instance the concomitant appearance of O-bound and N-bound nitrite ligands in frozen solutions in contrast to crystallographically ordered protein molecules where only the O-bound nitrito mode could be resolved.^31^,^32^ However, the exact reason for the origin of the two components in EPR spectra is currently not clear. Of all the ferriheme–NO_2_^–^ complexes found in the literature, NP4[NO_2_^–^] represents the most extreme case in the sense that besides the “normal” rhombic signal with *g* = (2.74, 2.42, 1.51), a spectrum with a “large *g*_max_” type of signal at *g*_max_ = 3.38 was also resolved (Fig. 1).^27^

The EPR spectrum directly reflects the electron configuration of LS d^5^ porphyrinates. There are two limiting electron configurations for such a system that give rise to three types of EPR spectra: (a) the normally observed (d_*xy*_)^2^(d_*xz*_,d_*yz*_)^3^ configuration, for which the EPR spectra may be either “large *g*_max_” (type I, usually with only a single observable *g* value >3.2, the so-called highly anisotropic low-spin (HALS) EPR signal) or normal rhombic (type II, with three observable *g*-values) in appearance; (b) the (d_*xz*_,d_*yz*_)^4^(d_*xy*_)^1^ configuration, for which an “axial” type of the EPR spectrum is observed (type III, 2.6 ≥ *g*_⊥_ > 2 > *g*_∥_).^33^ In type I hemes the energy difference between the d_*xz*_ and d_*yz*_ orbitals may be so small that they become near-degenerate and this leads to the tetragonal distortion of the d orbitals, showing a HALS EPR signal; as the energy gap between the two orbitals increases, the degeneracy is removed and a rhombic distortion is observed (type II). In a quite distinct ground state (type III), the d_*xz*_, d_*yz*_ orbitals are nearly degenerate and below the d_*xy*_ orbital in energy (see Fig. S1† for the splitting pattern of the LS d^5^ orbitals). In bis–His coordinated ferriheme systems the HALS species is related to the perpendicularly oriented His planes with respect to each other, whereas the rhombic signal is related to a more parallel orientation.^33^–^36^ However, as far as two different heme ligands—like in the case of cytochrome *c* (His and Met)^37^,^38^—are concerned, there is no clear cut difference between the large *g*_max_ and “normal” rhombic cases as a result of the relative ligand orientation. Rather, other factors have been suggested to modulate the electronic structures of the ferriheme center, such as heme ruffling and the bonding strength of the axial ligand.^37^–^41^ Therefore, it is not clear whether the HALS and rhombic signals observed in NP4[NO_2_^–^] are related to different NO_2_^–^ orientations with respect to the proximal His residue or if there are other contributing factors. A more detailed study is required to understand the molecular structural basis of the unique EPR spectrum observed in this case.

Herein, we report the first combined EPR spectroscopic and structural investigation on the nitrite complexes with various NP isoforms and NP4 variants. The outcome of the study promises to be highly relevant for elucidating the key factors contributing to the unprecedented nitrite dismutase activity of the nitrophorins.

## Results and discussions

### The influence of heme ruffling in different NPs

The heme out-of-plane distortion, *e.g.* ruffling, was reported to be able to affect the ground electronic configuration (and hence the line shape of the EPR spectrum) of the related LS d^5^ complex.^39^–^41^ It is well-known that NPs have highly ruffled hemes, and therefore, possible correlations between the observed HALS EPR signal of NP4[NO_2_^–^] and heme ruffling were probed. The EPR spectrum of the isoform, NP7[NO_2_^–^], showed no HALS signal (Fig. 1); rather, two sets of rhombic signals were resolved (LS1, *g* = 2.86, 2.34, 1.46; LS2, *g* = 2.78, 2.40, 1.46).^27^ Herein, we measured the EPR spectra of the nitrite complexes with other NPs, *e.g.* NP1 and NP2. The relative contribution of the two types of signals, *i.e.* HALS and rhombic LS, was estimated from the simulated spectra. All EPR simulation files are provided in the ESI (Fig. S2†). In all the cases, a small fraction of the high-spin signal at *g* ≅ (6.0, 6.0, 2.0) was observed, which is probably due to a water coordination complex. For NP4[NO_2_^–^], a HALS signal is clearly observed (≈30%). It can be seen that NP1[NO_2_^–^] also has a HALS signal with a similar ratio as in the case of NP4[NO_2_^–^], whereas NP2[NO_2_^–^] shows only two rhombic species with LS1, *g* = (2.95, 2.23, 1.41) and LS2, *g* = (2.70, 2.48, 1.44) (Fig. 1 and Table 1).

**Table 1:** Collection of the low-spin *g*-values of the nitrite complexes of NPs and related heme distortion

Protein complex	HALS or LS1			LS2			Relative spin contribution of HALS^*b*^	Heme distortion in NP[NH_3_] complexes	
	*g*_1_	*g*_2_	*g*_3_	*g*_1_	*g*_2_	*g*_3_		RMSD^*d*^ (0.01 Å)	PDB code
NP4[NO_2_^–^]^*c*^	3.38	—^*a*^	—^*a*^	2.74	2.42	1.51	≈30%	14.9	1X8P
NP1[NO_2_^–^]	3.33	—^*a*^	—^*a*^	2.72	2.41	1.50	≈30%	13.9	2NP1
NP2[NO_2_^–^]	2.95	2.23	1.41	2.70	2.48	1.44	≈0%	24.9	1EUO
NP7[NO_2_^–^]^*c*^	2.86	2.34	1.46	2.78	2.40	1.46	≈0%	18.5	4XMD

^*a*^“Large *g*_max_” type of spectrum, *g*_2_ and *g*_3_ are not observable.

^*b*^Data obtained from spectra simulations.

^*c*^From ref. 27.

^*d*^Calculated based on the heme core using the program Olex2.^42^

The degree of heme ruffling of the NP complexes was derived from the root-mean-square deviations (RMSD) from the heme mean plane as reported.^11^ An analysis of the heme ruffling of NPs reveals a seemingly clear trend of increasing ruffling with decreasing HALS species (Table 1). While there are relatively large differences in the degree of heme ruffling among different NPs (RMSD = 0.25 Å for NP2 *vs.* RMSD = 0.15 Å for NP4), it is not the case among the variants of a specific isoform, *e.g.* NP4. As we shall see later, the NP4 variants—although with similar heme ruffling situations—have very different EPR spectra for their nitrite complexes. Therefore, other factors may also contribute significantly to the origin of the HALS EPR signal in the nitrite complexes of NPs.

### The influence of the protonation state of the proximal His ligand in NP4 variants

**Scheme 2:**
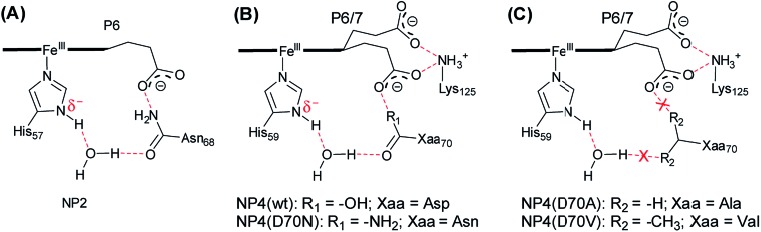
Schematic representation of the H-bonding network of the heme active site in the cases of NP2 ((A), PDB code 1EUO), NP4 and its variants ((B) and (C), derived from PDB code ; 1X8P). Note: this scheme is drawn on the basis of the X-ray structures and the H-bonds are indicated in red. The protonation state provided here is only tentative. The heme macrocycle is presented as a bold line. The heme propionates are indicated as P6, P7.

**Fig. 2:**
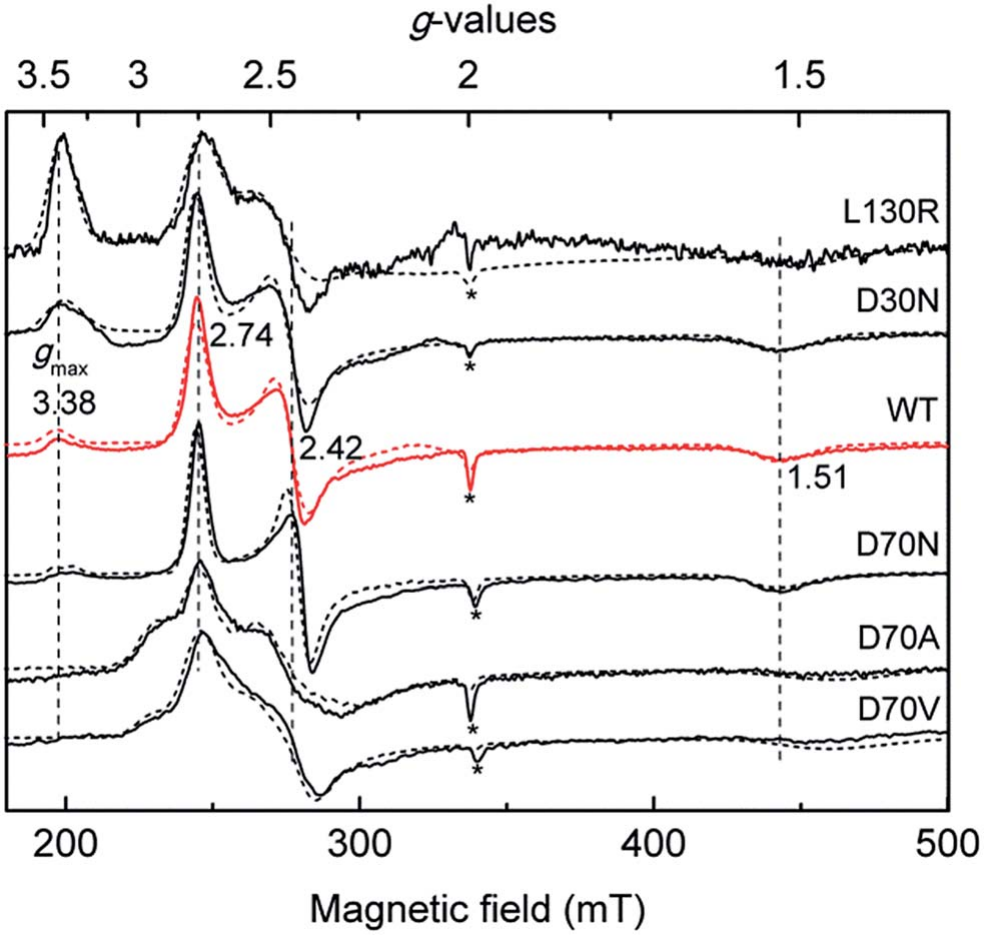
Comparison of the X-band EPR spectra of the nitrite complexes of NP4 variants recorded at 10 K, with the simulated spectra shown in dashed lines (see Fig. S2† for *g*-values used for the simulations). For comparison, the positions of the *g*-values of wt NP4[NO_2_^–^] are indicated with dashed vertical lines. Note that in all the cases a small fraction of the high-spin signal was observed (*), possibly due to a water-bound ferriheme complex.

It is widely accepted that the axial ligand can have a strong impact on the electronic structure of the heme iron center.^43^ Notably, as a common axial ligand the histidine side-chain can adopt multiple protonation states, *i.e.* neutral, N^ε^ protonated (histidinum), and N^δ^–H deprotonated (histidinate) (see Fig. S3† for details). The proximal heme ligand of NP2, *i.e.* His57, has a slight histidinate character (partially deprotonated) due to the weak H-bond network involving a water molecule, the Asn68 residue, and the P6 heme propionate (Scheme 2A).^44^ Interestingly, the Asn residue in NP2 is replaced by an Asp residue at the corresponding position in wt NP4 (Scheme 2B), where the bridging water molecule is conserved. Amino acid sequence alignment of NPs is provided in Fig. S4,† from which we can see that either an Asp or Asn is present at this position. Even more strikingly, the Asp70 residue in NP4 is part of an extended H-bonding network which involves the two heme propionates and the Lys125 residue. In this case, the ability to abstract the proton of the N^δ^H group of the axial His59 is much higher and it may lead to a more pronounced histidinate character of the His59 in NP4. In order to probe the possible consequences of the strengthened histidinate character on the electronic structure of the nitrite complex, a series of mutations have been introduced and the EPR spectra of the corresponding nitrite complexes recorded. Mutation of Asp70 to Asn in NP4—to mimic the NP2 environment—leads to a much lower ratio of the HALS species in the EPR spectrum of its nitrite complex, ≈16% (Fig. 2 and Table S1†). The presence of a HALS signal in this case suggests the contribution of the two heme propionates, which are now mediated by the newly introduced Asn residue (Scheme 2B). It is proposed that the negatively charged heme propionate would abstract the proton of the N^δ^H group of the axial His59 through the extended H-bonding network (Scheme 2B). A further mutation of Asp70 to either Ala or Val completely suppresses the HALS signal in the nitrite complex, *i.e.* only rhombic EPR signals can be observed (Fig. 2 and Table S1†). In both cases, the two heme propionates are decoupled from the rest of the H-bonding network and the His59 residue is essentially neutral, *i.e.* with the N^δ^H proton (Scheme 2C). It can be seen that these NP4 variants, although similar in heme ruffling deformation, exhibit a very different ratio of HALS signals in their nitrite complexes (Table S1†). The histidinate character of the axial His ligand is thus concluded to be an important factor for the appearance of HALS signals in the nitrite complexes of NPs.

**Fig. 3:**
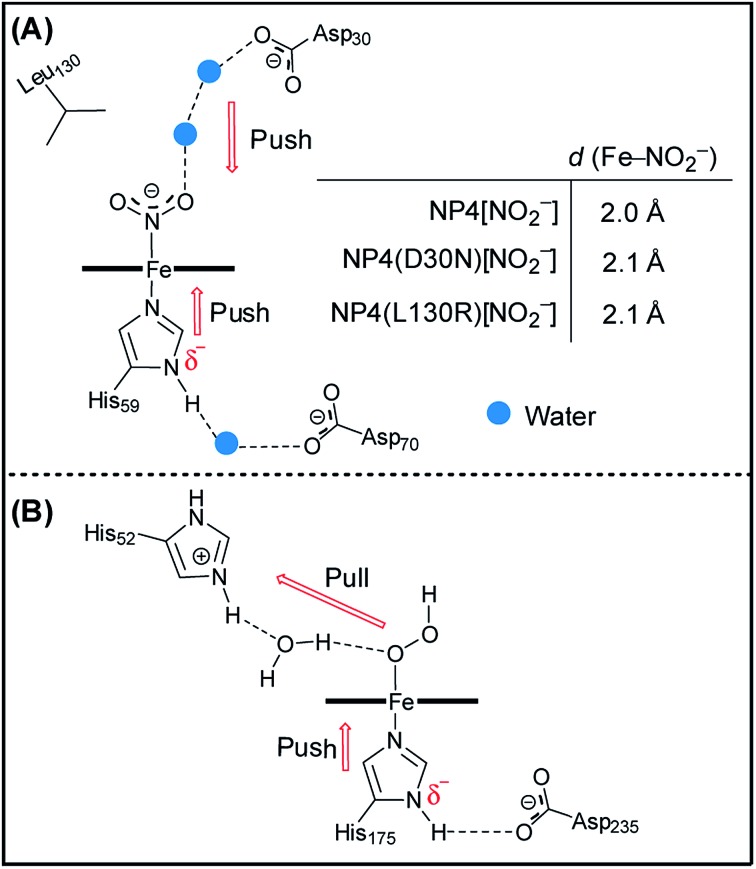
The active site (heme) environment of NP4[NO_2_^–^] ((A), PDB code: ; 3MVF) and a CcP with the modeled H_2_O_2_ substrate (compound 0) highlighting the push–pull mechanism ((B), adapted from ref. 50 and 51). The protonation state provided here is tentative, and for representation purposes.

Besides the proximal His ligand, the H-bond interactions between the axial ligand and the distal heme pocket residues may also have a significant impact on the electronic structure and reactivity of the heme complexes. The most well-known examples are the peroxidases where a distal His residue and the related H-bonds are essential for the polarization/cleavage of the O–O bond as they provide a “pull” effect on the distal side (Fig. 3B).^45^–^50^ Such an effect of the positively charged His52 in cytochrome *c* peroxidase (CcP) was proposed, by density functional calculations, to be mediated by a bridging water molecule.^50^,^51^ As mentioned in the introduction (see Scheme 1), unlike other protein–nitrite complexes, there is no amino acid residue in the distal heme pocket of the NP4[NO_2_^–^] complex which could form an H-bond directly to the nitrite ligand. However, two water molecules are present and are involved in an H-bonding network that connects the Asp30 residue to the nitrite ligand (compare Fig. 3A). Note that the Asp residue is highly conserved in the NP family (Fig. S4†). In a previous study, we showed that the NP4(D30N) variant has almost no nitrite dismutase activity, thus concluding that Asp30 is a key residue for its activity.^29^ Inspired by this result, we measured the EPR spectrum of the nitrite complex with NP4(D30N) (Fig. 2). Interestingly, it shows a significantly increased ratio of HALS species (≈60%) compared to that of the wt NP4[NO_2_^–^] (Table S1†). An even more pronounced HALS signal has previously been observed for another distal heme pocket variant—NP4(L130R)[NO_2_^–^], namely ≈72%.^29^

In a next step, we crystallized the NP4(D30N)[NO_2_^–^] complex and solved its structure (PDB code: ; 5HWZ, Fig. S5†). It shows that nitrite binds to the heme iron in an N-bound nitro form like in the cases of NP4[NO_2_^–^] and NP4(L130R)[NO_2_^–^] (Scheme 1). The two mutations D30N and L130R, however, dramatically interfere with the distal heme pocket in the sense that the Asp30···water···water···nitrite H-bonding network of the wt protein is disrupted in both cases (Fig. S6†). The increase of HALS species in the nitrite complexes of the two variants therefore suggests the contribution of the negatively charged Asp30. In contrast to the positively charged His52 residue in CcP, Asp30 in NP4 may exert a slight electron-donating (“push”) effect on the bound nitrite ligand. Such a “push” effect may weaken the “push” effect from the proximal histidinate residue (compare Fig. 3A). Consequently, removing the distal “push” effect—through either replacement by a neutral residue as is the case of the D30N mutation (Fig. S6C†) or introduction of a positively charged residue as is the case of the L130R mutation (Fig. S6B†)—may indirectly strengthen the “push” effect of the proximal histidinate residue. In doing so, NP4(D30N)[NO_2_^–^] and NP4(L130R)[NO_2_^–^] show clearly increased HALS species compared to that of the wt protein. In line with this is the EPR spectrum of NP4[NO_2_^–^] measured at pH 10.5 where a more pronounced HALS signal is observed (≈50%, Fig. S2J and Table S1†), here it is expected that the proximal His residue will deprotonate to a greater extent at a higher pH value. As such, it unambiguously assigns the histidinate character of the proximal His residue as a cause of the HALS EPR signal in NP4[NO_2_^–^].

### Implications for the nitrite dismutase activity

**Fig. 4:**
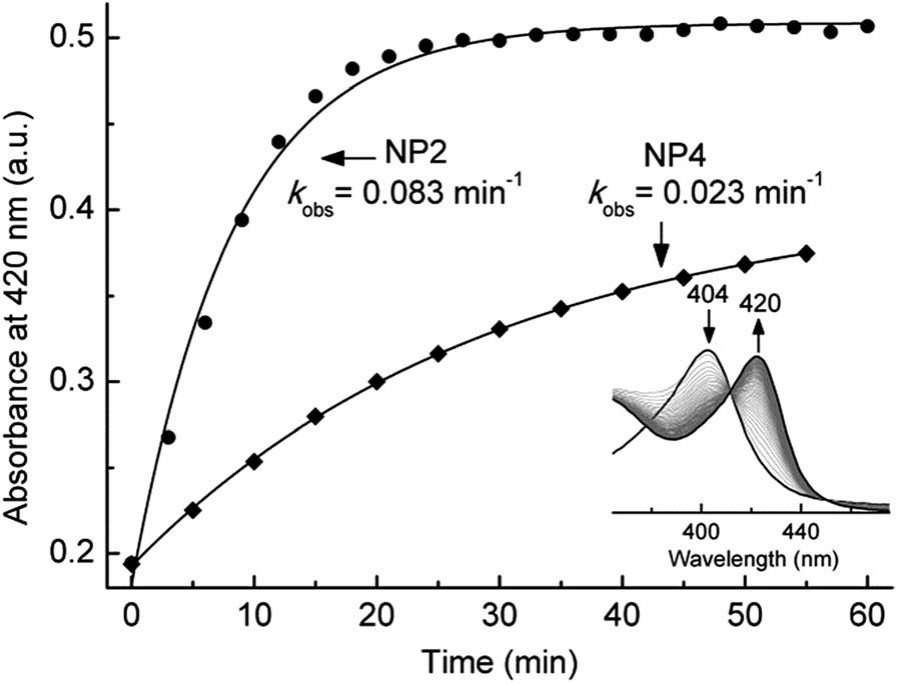
UV-vis absorbance changes at 420 nm—from which the *k*_obs_ was calculated—for the reaction of nitrite (20 mM) with NP2 (≈5 μM) and NP4 (≈5 μM) at 37 °C in 0.1 M phosphate buffer (pH 6.5). Insert: typical UV-vis absorption spectra for the reaction of NPs with nitrite.

Heme ruffling leads to overlap of the p_π_ orbitals of the heme nitrogens with the d_*xy*_ orbital of the metal, so that the electronic interaction between the porphyrin a_2u_(π) orbital and the metal d_*xy*_ orbital is switched on, as described in model heme complexes.^52^ This way ruffling helps to stabilize the (d_*xz*_,d_*yz*_)^4^(d_*xy*_)^1^ ground state of the LS d^5^ ferrihemes, which has been reported for model ferriheme complexes^53^,^54^ and in the CN^–^ complexes of NPs.^55^,^56^ Such an electronic configuration is proposed to be important for NP[NO] complexes, in which the unpaired electron of the NO ligand would be located on the NO moiety. Due to orbital orthogonality, the NO unpaired electron and the Fe^III^(d_*xy*_)^1^ of NPs may be coupled magnetically, leading to an Fe^III^(d_*xy*_)–˙NO configuration that is ready for NO release when needed.^11^ The EPR spectra of the nitrite complex of NPs—with either type I or type II signals—clearly indicate the (d_*xy*_)^2^(d_*xz*_,d_*yz*_)^3^ configuration of the ferriheme centers, at cryogenic temperatures, *e.g.* 10 K. However, the general trend of increasing ruffling with decreasing HALS species, *i.e.* smaller *g*-anisotropy, observed among the nitrite complexes with different NP isoforms seems to suggest a contribution of the (d_*xz*_,d_*yz*_)^4^(d_*xy*_)^1^ configuration. The most ruffled NP2[NO_2_^–^], with no HALS signal, may have more of the (d_*xz*_,d_*yz*_)^4^(d_*xy*_)^1^ configuration, whereas the less ruffled NP4[NO_2_^–^], with significantly more HALS signal, may adopt more of the (d_*xy*_)^2^(d_*xz*_,d_*yz*_)^3^ configuration. Adding to this is the fact that a (d_*xy*_)^2^(d_*xz*_,d_*yz*_)^3^ system may switch to a (d_*xz*_,d_*yz*_)^4^(d_*xy*_)^1^ system at elevated temperatures, as reported for model complexes^57^ and heme proteins.^56^,^58^ This supports the idea that strong ruffling of the NP[NO_2_^–^] complexes could stabilize a partial (d_*xz*_,d_*yz*_)^4^(d_*xy*_)^1^ ground electronic configuration, especially under physiological conditions, *e.g.* at 37 °C. It is worth noting that the coordination of NO_2_^–^, a reasonably strong π-acceptor ligand,^59^ may also contribute to stabilizing a (d_*xz*_,d_*yz*_)^4^(d_*xy*_)^1^ configuration of the Fe centers in the NP[NO_2_^–^] complexes. As mentioned, heme ruffling may help to stabilize the ferriheme NP[NO] complexes through a (d_*xz*_,d_*yz*_)^4^(d_*xy*_)^1^ configuration. Since this is a final product for the nitrite dismutation reaction catalyzed by NPs (eqn (1)), we speculated that NPs with a more ruffled heme should have a higher activity due to a comparable electron configuration, *i.e.* (d_*xz*_,d_*yz*_)^4^(d_*xy*_)^1^. In light of this, the nitrite dismutase activity of NP2 and NP4 was measured and compared in Fig. 4. It clearly shows that NP2 reacts much faster with nitrite (*k*_obs_ = 0.083 min^–1^*vs. k*_obs_ = 0.023 min^–1^ for NP4). As discussed, the heme ruffling contributes to the decrease of *g*-anisotropy in the EPR spectra of the nitrite complexes of NPs, whereas the HALS signal observed is due to a more planar heme. As far as the functional consequence is concerned, it is the ruffled heme which facilitates the nitrite dismutation reaction. It is worth noting in this context, that heme ruffling is known to stabilize the 3+ oxidation state of the iron center, *i.e.* lowers the heme Fe^III^/Fe^II^ redox potential, in a number of ferriheme proteins.^58^,^60^–^62^ We have shown that a lower redox potential of the heme iron center is the key to NPs' nitrite dismutase activity.^63^ It can be rationalized that NPs, by adopting a ruffled heme conformation, have a much lower redox potential relative to other hemeproteins, *e.g.* metMb (≈300 mV more positive),^11^ which is essential for their unique reactivity with nitrite.

As described above, the significantly increased ratio of HALS species in the nitrite complexes of the two NP4 variants, namely NP4(D30N) and NP4(L130R), has been attributed to the presence of a partially deprotonated proximal His ligand, *i.e.* a histidinate. The nitrite dismutase activity in the two variants has been shown to have almost disappeared,^29^ apparently not because of heme ruffling changes (Table S1†). The importance of H-bonding to the proximal His residue in controlling heme protein activity has long being discovered, with the Asp–His–Fe triad of CcP being one of the most extensively studied systems.^45^–^50^ Therefore, a possible role of the histidinate character of the proximal His residue in modulating the nitrite dismutase activity of NPs is suspected. Upon deprotonation, the resulting imidazolate moiety of the proximal His is expected to be a stronger field ligand than the neutral imidazole.^46^,^64^,^65^ The negative charge on the imidazolate, *via* increased ligand field strength, is generally central in modulating many of the reactivity and ligand-binding properties of heme proteins. In the cases of the nitrite complexes of NP4(L130R) and NP4(D30N), a negatively charged proximal histidinate—resulting from the perturbation of the H-bonding network in the distal heme pocket—would lead to a weakened Fe–N_nitrite_ coordination compared to that of the wt protein due to the trans effect or electron–electron (anion–anion) repulsion. In line with this are the slightly larger Fe–N_nitrite_ bond distances observed in the complexes of NP4(L130R)[NO_2_^–^] (2.1 Å) and NP4(D30N)[NO_2_^–^] (2.1 Å) compared to that of NP4[NO_2_^–^] (2.0 Å) (compare Fig. 3). As such, the weakly bound initial nitrite complexes could contribute to the much lower nitrite dismutase activity observed for the two variants. Moreover, it was demonstrated that the Fe center of a porphyrin[Fe^III^–NO_2_^–^] complex is extremely electron deficient and to relieve the “hunger for electron density” is a major driving force for many of its reactivities.^59^ In this regard, a negatively charged proximal histidinate ligand is expected to push more electron density into the Fe center, *e.g.* of the NP4(L130R)[NO_2_^–^] and NP4(D30N)[NO_2_^–^] complexes, thereby, satisfying its electron demand to some extent. Consequently, the tendency to relieve the “hunger for electron density”, *i.e.* to react, is diminished in these complexes, as observed in a previous study.^29^ On the contrary, in the cases of other NP4 variants where Asp70 is mutated to either Val or Ala and the proximal His residue is essentially neutral (compare Scheme 2C), the corresponding nitrite dismutase activity is higher compared to that of the wt NP4 (*e.g. k*_obs_ = 0.07 min^–1^ for NP4(D70A), Fig. S7†). As a result, it establishes that a histidinate character of the proximal His residue contributes significantly to the lowering of the nitrite dismutase activity of the NPs. It is well-known that the nitrite dismutation catalyzed by NPs is strongly pH-dependent, with a much smaller reaction rate at high pH, *e.g.* >7.5.^15^ From the discussion above, it can be deduced that the smaller activity is due to a stronger histidinate character of the proximal His residue in this case.

## Conclusions

The origin of the unprecedented HALS EPR signals of the NP[NO_2_^–^] complexes has been extensively investigated in this study. It is established that both heme conformation, *e.g.* ruffling, and the protonation state of the proximal His residue can affect the EPR spectra of the corresponding nitrite complexes. More importantly, both factors contribute to the nitrite dismutase activity of NPs. The highly ruffled hemes in NPs, and the concomitantly decreased Fe^III^/Fe^II^ redox potentials as compared to those in most other heme *b* proteins or model complexes, place them in a unique position to facilitate nitrite dismutation. However, heme ruffling or low redox potential is not the only reason; otherwise other ferriheme *b* proteins, *e.g.* the peroxidases which are known to have a low redox potential,^66^ may also catalyze the reaction. As discussed, the histidinate character of the proximal His residue, or more precisely the extended H-bonding network at the heme active site, also plays a pivotal role in determining the nitrite dismutase activity in NPs. In this regard, a neutral proximal His residue proves to be more reactive (discussed above), which is probably the major reason why such activity is not found in peroxidases—where hemes are also highly distorted^67^ and the Fe^III^/Fe^II^ redox potentials can be as low as those in NPs (+5 to –310 mV for peroxidases^66^*vs.* –259 to –335 mV for NPs^68^,^69^). Similarly, NP4(D30N) was reported to have a lower redox potential than that of the wt NP4 (–429 mV for NP4(D30N) *vs.* –384 mV for wt NP4 at pH 5.0),^70^ however, it cannot react with nitrite, which clearly argues for the role of the H-bonding network of NPs in determining the nitrite dismutase activity.

In summary, the key factors that facilitate the nitrite dismutase reaction of NPs have been established to be the heme ruffling and an extended H-bonding network at the active site which helps to keep the proximal His residue neutral. We believe the structural features revealed herein would be very helpful in identifying other heme proteins, especially in mammals, which may have similar activity. Moreover, it can serve as a guideline for the design of model heme complexes or heme proteins with (enhanced) nitrite dismutase activity.

## Experimental

### Materials

Stock solutions of NaNO_2_ were prepared freshly before use and the concentration was photometrically calibrated (*ε*_210 nm_ = 5380 M^–1^ cm^–1^).^71^ All other reagents were of the highest grade commercially available and used as received. Expression plasmids for the preparation of the protein variants NP4(D70→A/V/N) were commercially derived from the expression plasmid of wt NP4 (GenScript Inc.).

### Protein preparation

NP4 and its variants NP4(D30N), NP4(D70V), NP4(D70A), NP4(D70N), and NP4(L130R) as well as the wt forms of NP1, NP2, and NP7 were recombinantly expressed in the *Escherichia coli* strain BL21(DE3) (Novagen) and purified and reconstituted as was previously described.^13^,^72^ Protein preparations were routinely analyzed by SDS-PAGE to be >90% pure. The proteins were subjected to MALDI Q-TOF MS to confirm the correct molecular masses accounting for two Cys–Cys disulfides. Proteins were kept frozen in 200 mM NaOAc/AcOH (pH 5.5), 5% (w/v) glycerol until use.

### EPR spectroscopy

The solvent of the protein was exchanged to 100 mM HEPES/NaOH (pH 7.2), 25% (w/v) glycerol and the concentration was adjusted to ≈300 μM. Upon mixing with 300 mM of NaNO_2_, the protein samples were incubated at room temperature for a few minutes. The samples were then quickly transferred into a 3 mm quartz tube and then rapidly frozen in liquid N_2_ where they were kept until measurement. Continuous wave (cw)-EPR spectra were recorded on a Bruker ESP-380E spectrometer at X-band frequency equipped with a gas-flow cryogenic system using a liquid He cryostat from Oxford Inc. (ESR 935) and an ITC4 temperature controller. Spectra were recorded at 10 K with a microwave power of 1 mW, a field modulation of 100 kHz, and a modulation amplitude of 0.5 mT. The obtained EPR spectra were simulated with the program GEESTRAIN5 V 1.0.^73^

### X-ray crystallography

Protein crystals were obtained from 10 mg mL^–1^ of NP4(D30N) in water using the vapor-diffusion method upon mixing with an equal volume of 2.8 M ammonium phosphate (pH 7.4). The crystals were soaked for 10 min on ice in 3.2 M potassium phosphate (pH 7.4) containing 0.3 M KNO_2_ and afterwards in 3.2 M potassium phosphate (pH 7.4), 15% glycerol as a cryo protectant. Afterwards, the crystals were immediately frozen in liquid N_2_ and kept there until the measurement. A diffraction data set was collected at 100 K using the beamline BL14.2 at BESSYII (Berlin, Germany). The data set was processed with XDS^74^ and CCP4.^75^ The molecular-replacement method was applied using MOLREP^76^ and an initial model from NP4 (PDB code ; 3MVF).^27^ Model building and refinement were carried out using WINCOOT^77^ and PHENIX,^78^ respectively. The bond/angle parameters of the ligands on iron were refined with restrain. Data collection and refinement statistics are summarized in Table S2.† The stereochemical properties were checked by RAMPAGE.^79^

## Supplementary Material

Supplementary information
