# Supplementary material for: Elucidation of the heme active site electronic structure affecting the unprecedented nitrite dismutase activity of the ferriheme b proteins, the nitrophorins
Source: Chem Sci. 2016 Apr 25;7(8):5332–40. doi: 10.1039/c6sc01019a (PMC6020753; doi:10.1039/c6sc01019a)
Supplement: Supplementary file 1 [file SC-007-C6SC01019A-s001.pdf]

Electronic Supplementary Material (ESI) for Chemical Science.
This journal is © The Royal Society of Chemistry 2016

ELECTRONIC SUPPLEMENTARY INFORMATION

Elucidation of the heme active site electronic structure affecting the unprecedented nitrite dismutase activity of the ferriheme *b* proteins, the nitrophorins

Chunmao He,* Hideaki Ogata, and Wolfgang Lubitz*

Max Planck Institute for Chemical Energy Conversion, Stiftstrasse 34-36, D-45470, Mülheim an der Ruhr, Germany

*To whom correspondence should be addressed.

C.H. (chunmao82@gmail.com)

W.L. (wolfgang.lubitz@cec.mpg.de)

Fig. S1 The d orbital splitting patterns of Fe(III) low spin complexes.[a]

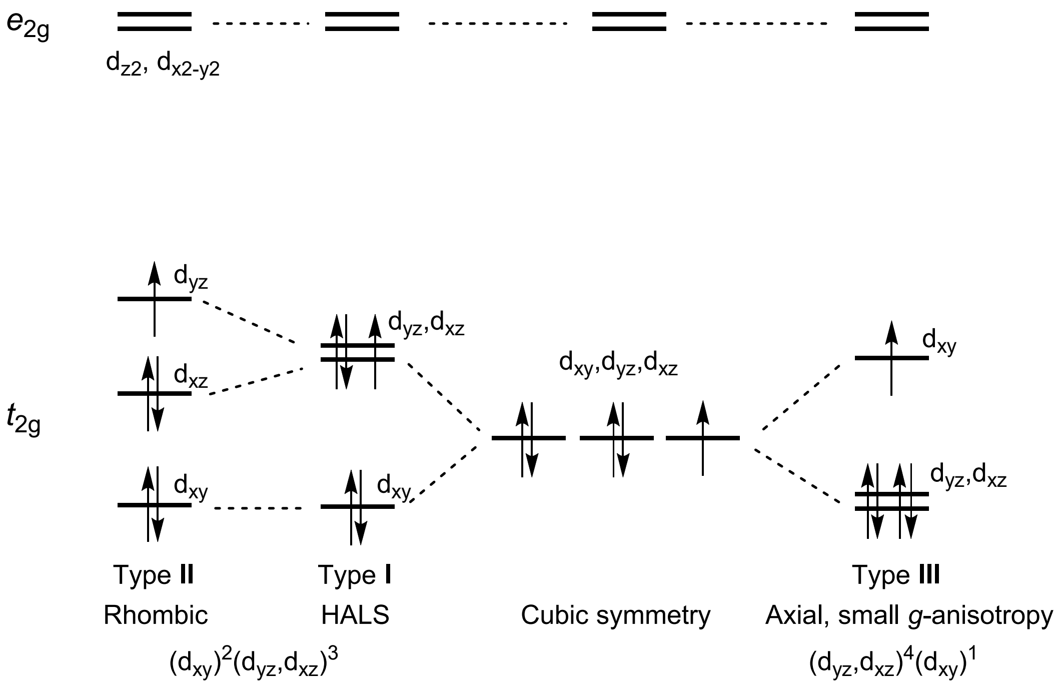

[a]Adapted from 1), M. Nakamura, *Coord. Chem. Rev.* 2006, **250**, 2271 and 2), G. Zoppellaro, *et al. J. Am. Chem. Soc.* 2008, **130**, 15348.

Fig. S2 Simulation of the EPR spectra of the nitrite complexes of nitrophorins at pH 7.4 unless noted otherwise (showing only the high field region; the residual high-spin signal is indicated by an asterisk). The simulated rhombic signal is shown in either blue or green and the HALS signal is shown in red; the g-values used for the simulations are summarized in the table to the right.

(A) NP4(L130R)[NO_2^-]

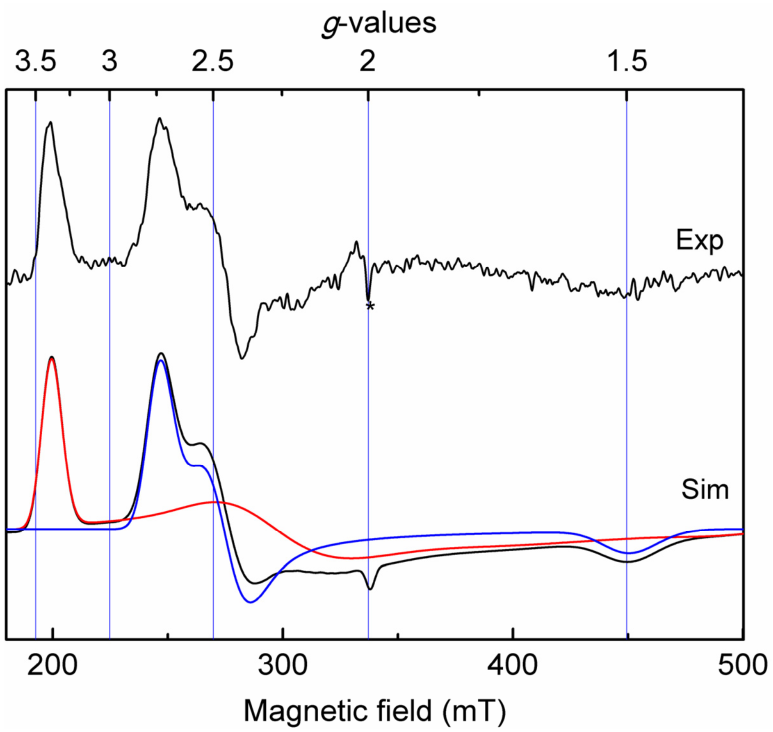

HALS			LS2		
g_1	g_2	g_3	g_1	g_2	g_3
3.32	2.20	0.60	2.70	2.42	1.49

(B) NP4(D30N)[NO_2^-]

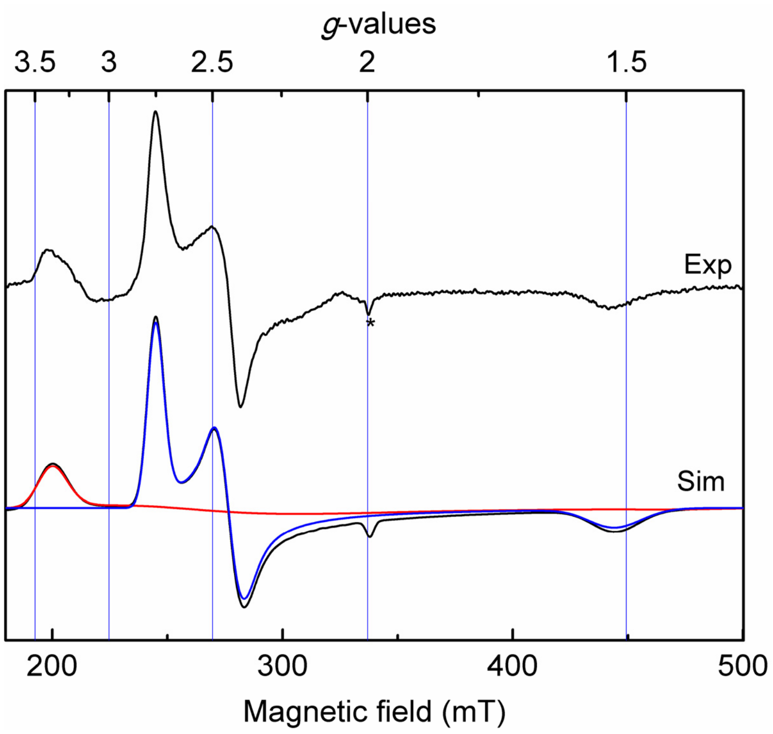

HALS			LS2		
g_1	g_2	g_3	g_1	g_2	g_3
3.30	2.05	0.62	2.72	2.41	1.51

(C) NP4[NO_2^-]

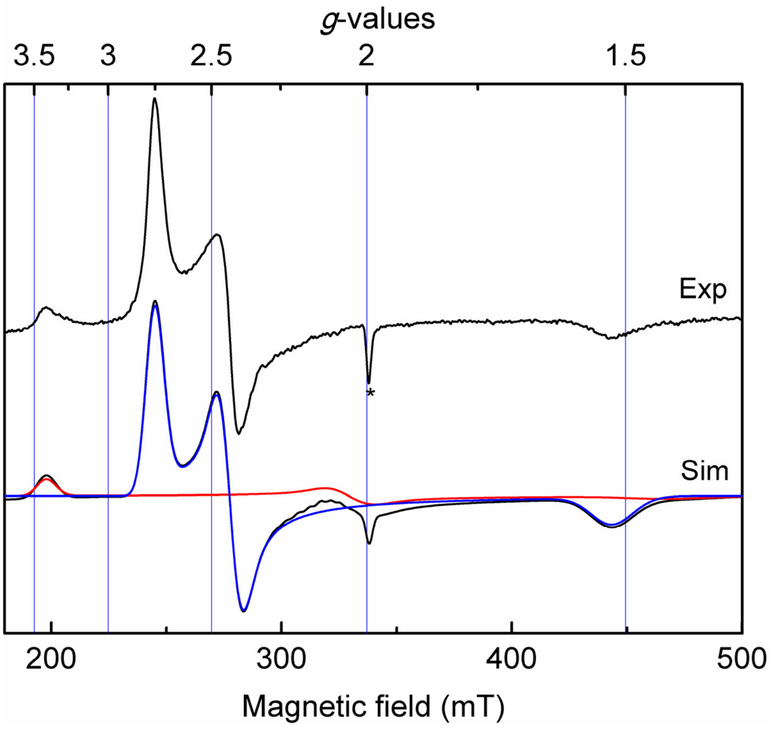

HALS			LS2		
g_1	g_2	g_3	g_1	g_2	g_3
3.38	2.03	0.62	2.74	2.42	1.51

(D) NP1[NO_2^-]

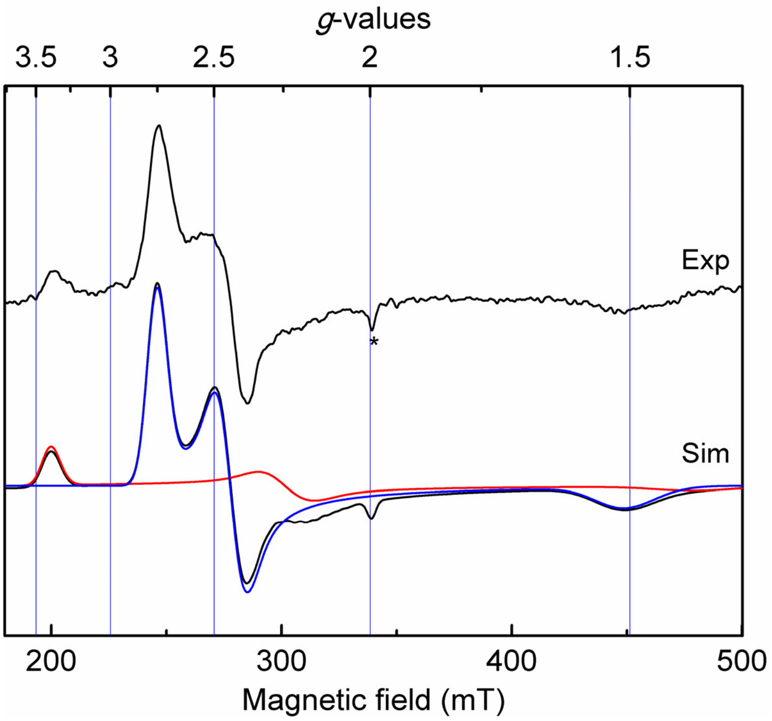

HALS			LS2		
g_1	g_2	g_3	g_1	g_2	g_3
3.33	2.22	0.55	2.72	2.41	1.50

(E) NP4(D70N)[NO_2^-]

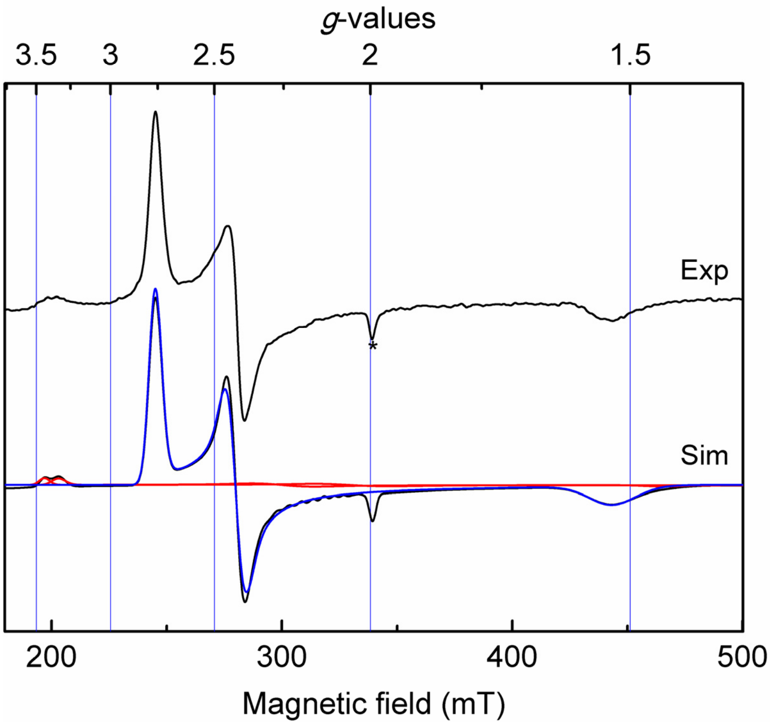

HALS			LS2		
g_1	g_2	g_3	g_1	g_2	g_3
3.38	2.03	0.50	2.73	2.40	1.52
3.28	2.23	0.65			

(F) NP4(D70V)[NO_2^-]

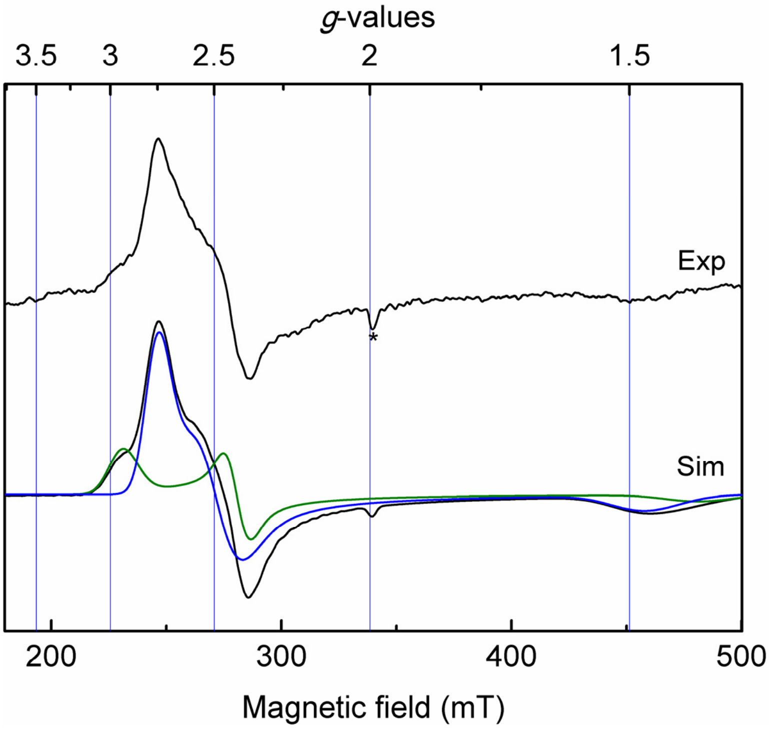

LS1			LS2		
g_1	g_2	g_3	g_1	g_2	g_3
2.89	2.39	1.40	2.71	2.46	1.47

(G) NP4(D70A)[NO_2^-]

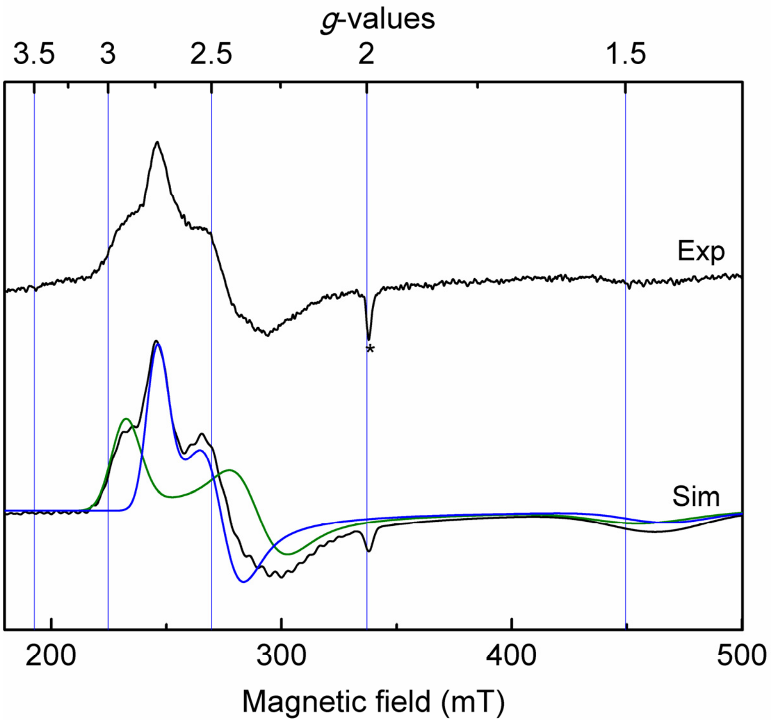

LS1			LS2		
g_1	g_2	g_3	g_1	g_2	g_3
2.86	2.30	1.40	2.71	2.43	1.43

(H) NP7[NO_2^-]

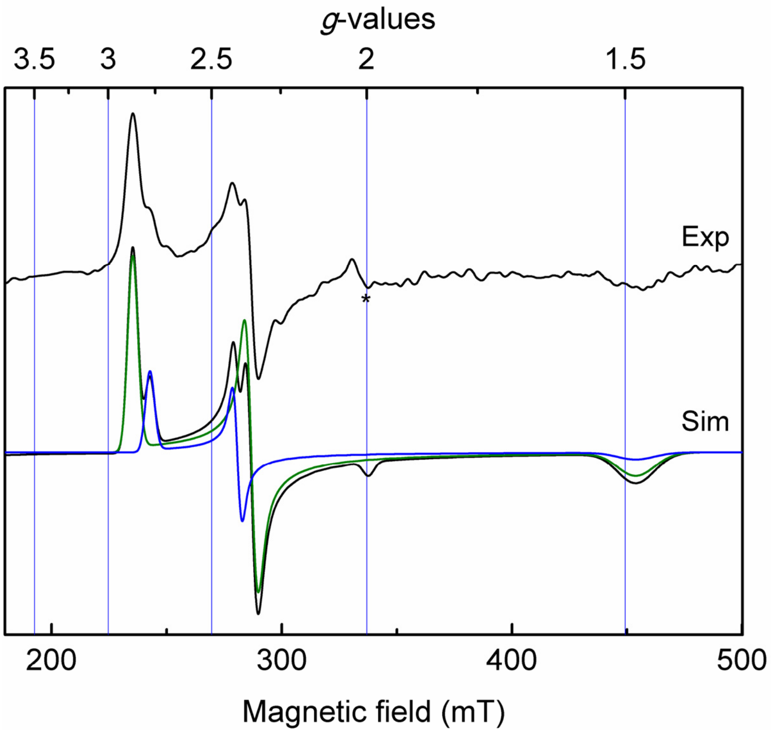

LS1			LS2		
g_1	g_2	g_3	g_1	g_2	g_3
2.86	2.34	1.46	2.78	2.40	1.46

(I) NP2[NO_2^-]

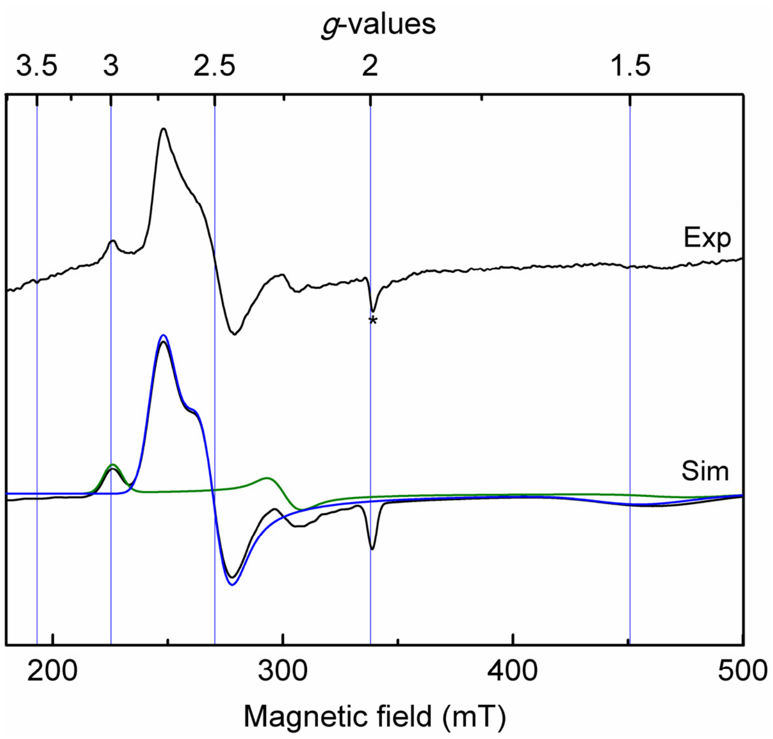

LS1			LS2		
g_1	g_2	g_3	g_1	g_2	g_3
2.95	2.23	1.41	2.70	2.48	1.44

(J) NP4[NO_2^-] at pH 10.5

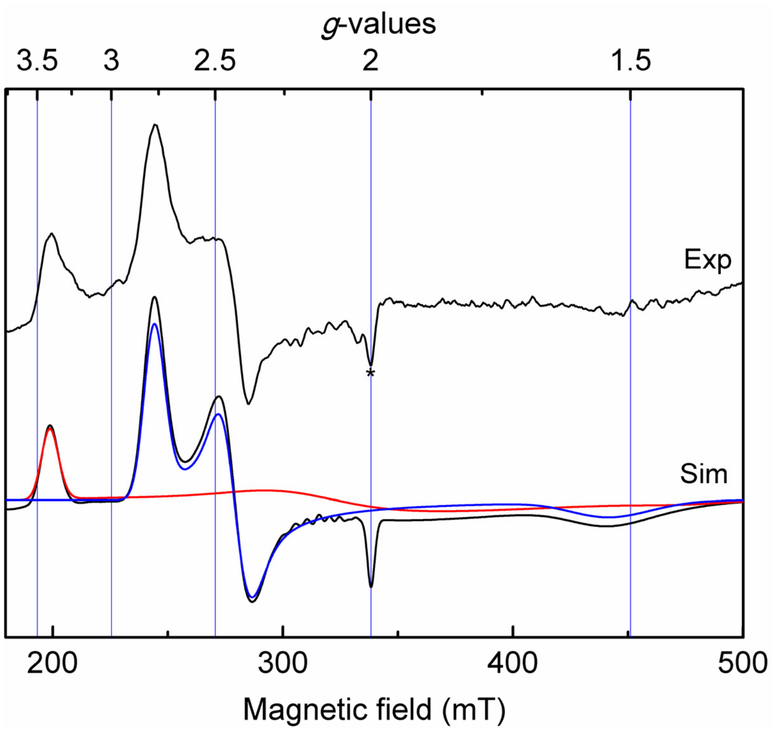

HALS			LS2		
g_1	g_2	g_3	g_1	g_2	g_3
3.40	2.05	0.66	2.77	2.42	1.52

Fig. S3 Possible protonation states of the histidine side-chain.[a]

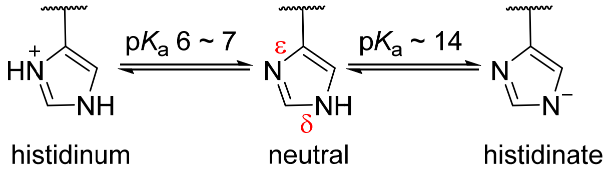

[a]Nomenclature of the N-atoms of the His side-chain are indicated in red, pK_a values are taken from: A. Barth, *Prog. Biophys. Mol. Biol.*, 2000, **74**, 141.

Fig. S4 Amino acid sequence alignment of *R. prolixus* NP1 (Swiss-Port entry Q26239), NP2 (Swiss-Port entry Q26241), NP3 (Swiss-Port entry Q94733), NP4 (Swiss-Port entry Q94734), and NP7 (Swiss-Prot entry Q6PQK2). The total sequence identity among all 5 protein sequences amounts to 33% (indicated by an asterisk). The proximal His is indicated by '#'. The initial Met residue (in grey) of the recombinantly expressed proteins of NP1, NP2, NP3, and NP7 is not present in the mature proteins *in vivo*. Residues of relevance to this study are highlighted in color.

```
     0         10         20         30         40         50         60
NP1: M    KCTKNAL AQTGFNKDKY FNGDVWYVTD YLDLEPDDVP KRYCAALAAG TASGKLKEAL (57)
NP2: M    DCSTNIS PKQGLDKAKY FSG-KWYVTH FLDKDP-QVT DQYCSSFTPR ESDGTVKEAL (55)
NP3: M    DCSTNIS PKKGLDKAKY FSG-TWYVTH YLDKDP-QVT DPYCSSFTPK ESGGTVKEAL (55)
NP4:      ACTKNAI AQTGFNKDKY FNGDVWYVTD YLDLEPDDVP KRYCAALAAG TASGKLKEAL (57)
NP7: M LPGECSVNVI PKKNLDKAKF FSG-TWYETH YLDMDP-QAT EKFCFSFAPR ESGGTVKEAL (58)
           *  *         * *  * *  ** *   **  *        *          *  ****

        #      70         80         90        100        110        120
NP1:   YHYDPKTQDT FYDVSELQEE SPG-KYTANF KKVEKNGNVK VDVTSGNYYT FTVMYADDSS (116)
NP2:   YHYNANKKTS FYNIGEGKLE SSGLQYTAKY KTVDKKKAVL KEADEKNSYT LTVLEADDSS (115)
NP3:   YHFNSKKKTS FYNIGEGKLG SSGVQYTAKY NTVDKKRKEI EPADPKDSYT LTVLEADDSS (115)
NP4:   YHYDPKTQDT FYDVSELQVE SLG-KYTANF KKVDKNGNVK VAVTAGNYYT FTVMYADDSS (116)
NP7:   YHFNVDSKVS FYNTGTGPLE SNGAKYTAKF NTVDKKGKEI KPADEKYSYT VTVIEAAKQS (118)
       **         **         *    ***     * *              **  **  *   *

              130        140        150        160        170        180
NP1:   ALIHTCLHKG NKDLGDLYAV LNRNKDTNAG DKVKGAVTAA SLKFSDFIST KDNKCEYDNV (176)
NP2:   ALVHICLREG SKDLGDLYTV LTHQKDAEPS AKVKSAVTQA GLQLSQFVGT KDLGCQYD-D (174)
NP3:   ALVHICLREG PKDLGDLYTV LSHQKTGEPS ATVKNAVAQA GLKLNDFVDT KTLSCTYD-D (174)
NP4:   ALIHTCLHKG NKDLGDLYAV LNRNKDAAAG DKVKSAVSAA TLEFSKFIST KENNCAYDND (176)
NP7:   ALIHICLQED GKDIGDLYSV LNRNKNALPN KKIKKALNKV SLVLTKFVVT KDLDCKYD-D (177)
       ** * **     ** **** * *   *         * *           *  * *   * **

NP1:   SLKSLLTK (184)
NP2:   QFTSL    (179)
NP3:   QFTSM    (179)
NP4:   SLKSLLTK (184)
NP7:   KFLSSWQK (185)
          *
```

Fig. S5 X-ray crystal structure of NP4(D30N) in complex with nitrite, highlighting the active site structure (PDB code: 5HWZ).

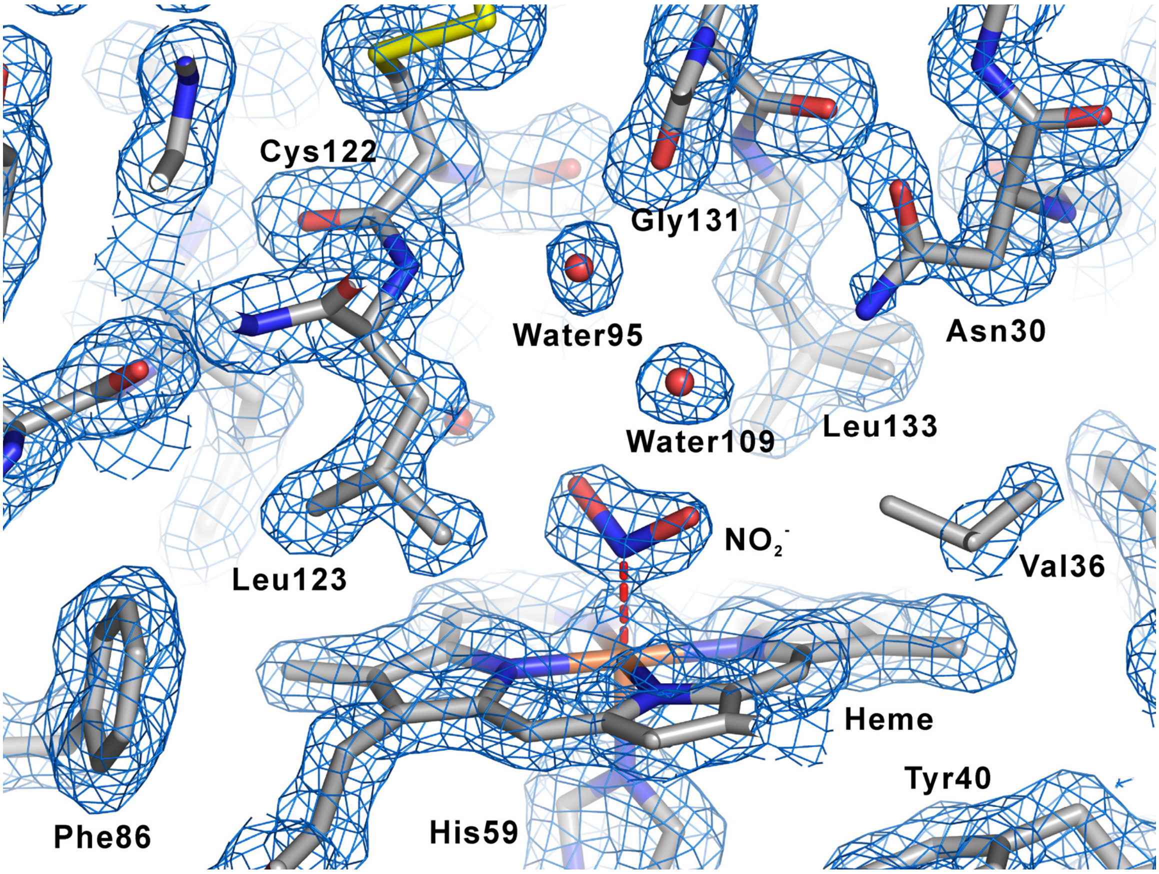

Fig. S6 Comparison of the distal heme pocket of the nitrite complexes of wt NP4 (A), NP4(L130R) (B), and NP4(D30N) (C). Shown in red dotted lines are the H-bonds with the corresponding distances indicated (Å).

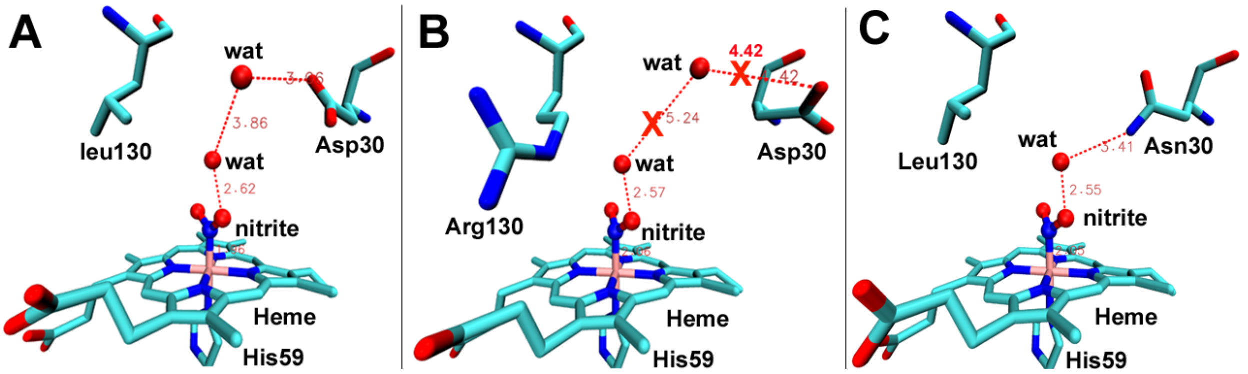

Fig. S7 UV–vis absorbance changes at 420 nm for the reaction of NP4(D70A) with nitrite at 37 °C, other experimental conditions are the same as described in Fig. 4. The k_{obs} is calculated from an exponential fitting (black line).

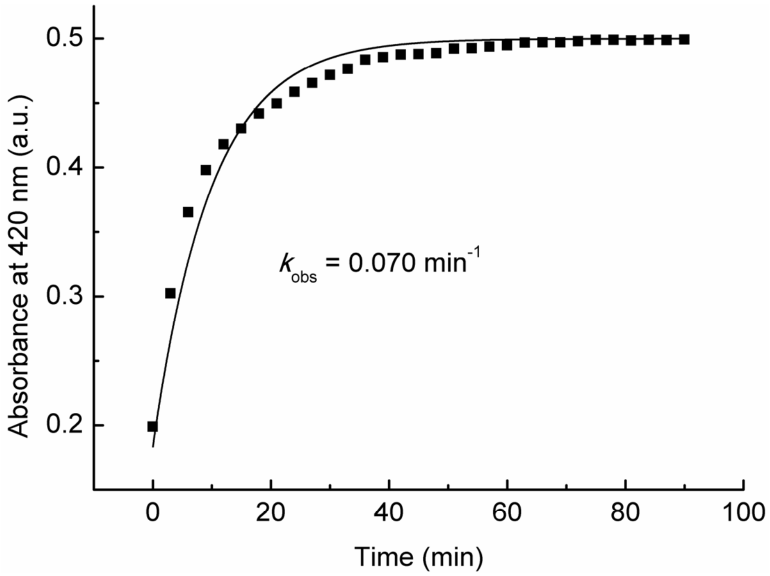

Table S1. Low-spin *g*-values of the nitrite complexes of NP4 variants and related heme distortion.

Protein complex	HALS or LS1			LS2			Relative spin contribution of HALS[b]	Heme distortion in $NP[NH_3]$ complexes	
	g_1	g_2	g_3	g_1	g_2	g_3		RMSD (0.01 Å)	PDB code
$NP4[NO_2^-]$	3.38	--[a]	--[a]	2.74	2.42	1.51	≈ 30%	14.9	1X8P
$NP4[NO_2^-]$, pH 10.5	3.40	--[a]	--[a]	2.77	2.42	1.52	≈ 50%	--	--
NP4(L130R) $[NO_2^-]$	3.32	--[a]	--[a]	2.70	2.42	1.49	≈ 72%	14.7	3TGA
NP4(D30N) $[NO_2^-]$	3.30	--[a]	--[a]	2.72	2.41	1.51	≈ 60%	17.0	1SXY
NP4(D70N) $[NO_2^-]$	3.38<br>3.28	--[a]<br>--[a]	--[a]<br>--[a]	2.73	2.40	1.52	≈ 16%	13.4	[c]
NP4(D70A)$[NO_2^-]$	2.86	2.30	1.40	2.71	2.43	1.43	≈ 0%	12.6	[c]
NP4(D70V) $[NO_2^-]$	2.89	2.39	1.40	2.71	2.46	1.47	≈ 0%	14.5	[c]

[a] "Large g_{max}" type of spectrum, g_2 and g_3 are not observable; [b]data obtained from spectra simulations; [c]unpublished data

Table S2. NP4(D30N)[NO_2^-]: Data collection and refinement statistics

	NP4(D30N)[NO_2^-]
PDB entry	5HWZ
Data Collection	
wavelength(Å)	0.91841
Space group	*C*2
Unit cell parameters	
a(Å)	69.78
b(Å)	43.11
c(Å)	52.62
β(°)	94.5
resolution(Å) [a]	26.23-1.45 (1.49-1.45)
no. of observed reflections	102441
no. of unique reflections	27719
R_{merge} [a]	0.040 (0.309)
completeness(%) [a]	99.7 (99.5)
$\langle I/\sigma(I)\rangle$ [a]	19.5 (4.3)
Refinement	
resolution range(Å)	26.2-1.45
R(%)	14.9
R_{free}(%)	18.0
no. of residues	184
no. of solvent molecules	118
ligand	NO_2^-
rmsd[b] for bond lengths(Å)	0.006
rmsd[b] for bond angles(°)	1.06
Ramachandran plot	
favored region(%)	99.5
allowed region(%)	0.5
outlierregion(%)	0.0
average *B* factor(Å^2)	
protein	17.1
ligand(heme)	17.6
ligand	24.8
solvent	24.5

[a] Numbers in parentheses represent the values for the highest-resolution shell.
[b] Root-mean-square deviation.
